# Follow-up–dependent calendar-period associations with cardiac mortality after lung cancer radiotherapy: a competing-risks SEER analysis

**DOI:** 10.1515/med-2026-1509

**Published:** 2026-08-25

**Authors:** Na Zhang, Siyu Chen, Jingyi Ai, Chaoxing Liu, Ye Zhou, Yajing Wu, Jun Wang

**Affiliations:** Department of Radiation Oncology, The Fourth Hospital of Hebei Medical University, Hebei Clinical Research Center for Radiation Oncology, Shijiazhuang, China; Department of Radiation Oncology, Shijiazhuang People’s Hospital, Shijiazhuang, China; Department of Oncology, Hebei General Hospital, Shijiazhuang, China

**Keywords:** competing risks, Fine–Gray model, subdistribution hazard, registry study, follow-up duration, cardio-oncology

## Abstract

**Objectives:**

Calendar-period comparisons of cardiac mortality after thoracic radiotherapy can be distorted by competing cancer death and by unequal follow-up, because more recently diagnosed patients are observed for less time. We aimed to determine whether a reported recent-era decline in cardiac mortality after lung radiotherapy reflects changing practice or analytic artifact.

**Methods:**

We identified 56,241 patients with lung and bronchus cancer diagnosed in 2009–2021 in the SEER database (28,246 beam radiotherapy without surgery; 27,995 surgery without radiotherapy; chemotherapy-recorded patients excluded). Cardiac death was the event of interest and lung cancer death the competing event. In each cohort we estimated Fine–Gray subdistribution hazard ratios (sHRs) for diagnosis period under (i) full follow-up, (ii) full follow-up restricted to diagnoses through 2018, and (iii) a fixed 36-month window for that same 2009–2018 cohort. We additionally estimated period sHRs across fixed windows of 12–60 months, repeated all models treating other-cause deaths as competing events and using cause-specific Cox regression, and tested the proportional-subdistribution-hazards assumption.

**Results:**

In the radiotherapy cohort, more recent diagnosis was associated with a lower subdistribution hazard of cardiac death under full follow-up (sHR 0.761; 95 % CI 0.678–0.854; p<0.001), but the association was not significant at any equalized follow-up window (12-month sHR 0.864; 24-month 0.918; 36-month 0.994 [0.853–1.157]; 48-month 0.944; 60-month 1.026; all p≥0.14), and the period effect was non-proportional over follow-up time (p=0.003). The attenuation persisted when other-cause deaths were treated as competing events (36-month sHR 1.000) and under cause-specific regression (36-month HR 0.898; p=0.167), and median follow-up differed markedly by period (108 vs. 38 months). By contrast, the surgery cohort retained a significantly lower recent-period estimate through 36 months under the same matched-window analysis (restricted-full sHR 0.734; 36-month sHR 0.770; p=0.017), attenuating only at 48–60 months.

**Conclusions:**

A recent-period reduction in the surgery cohort persisted through a 36-month matched window, showing that matched-window analysis does not mechanically null a calendar-period signal; by contrast, the radiotherapy cohort showed no such association at any comparable follow-up. Registry data therefore do not support attributing a cardiac-mortality decline to changes in radiotherapy practice, and temporal registry comparisons must use comparable follow-up windows, account for competing death, and verify proportionality before such attribution.

## Introduction

Lung cancer remains a major global health burden and a leading cause of cancer mortality [1], 2]. Although prognosis remains poor for many patients, advances in screening, staging, local therapy, systemic treatment, and supportive care have improved survival in selected populations. With longer survival, noncancer outcomes, including cardiovascular morbidity and mortality, are increasingly relevant.

External-beam radiotherapy is used across several lung cancer settings, including medically inoperable early-stage disease, locally advanced disease, selected postoperative settings, oligometastatic disease, and palliation [1]. Improvements in treatment planning and delivery can increase dose conformality and may limit irradiation of adjacent organs, including the heart. Whether changes in radiotherapy practice have translated into lower cardiac mortality at the population level remains uncertain [3].

Earlier thoracic organ-at-risk guidance provided detailed contouring and dose recommendations for several noncardiac structures [4], whereas evidence-based constraints for the heart and individual cardiac substructures were less well established [3]. Cardiac exposure received greater attention after RTOG 0617, in which dose escalation from 60 to 74 Gy failed to improve overall survival and was associated with worse outcomes [5]. Subsequent studies linked whole-heart exposure to cardiac events [6], cardiac dose and pre-existing cardiac disease to mortality [7], and exposure of selected cardiac subregions to overall survival [8]. However, much of this evidence has been derived from secondary dosimetric analyses and retrospective studies rather than prospective trials designed specifically to evaluate cardiac mortality.

Large population-based cancer registries allow uncommon outcomes to be examined across broad patient populations, but both treatment-group and calendar-period comparisons require caution. Patients selected for radiotherapy and surgery differ in age, disease stage, comorbidity, functional status, treatment indication, and expected survival. Lung cancer death may also occur before a later cardiac death can be observed and therefore represents an important competing event. A previous population-based study reported lower cardiac mortality in more recent treatment eras among patients with locally advanced non-small-cell lung cancer [9]. However, patients diagnosed more recently have inherently shorter potential follow-up and less opportunity for late cardiac deaths to be recorded. Calendar-period associations should therefore be evaluated using comparable follow-up windows before they are attributed to changes in treatment.

These methodological concerns prompted us to examine whether the diagnosis-period association in the beam radiotherapy cohort was sensitive to the available follow-up horizon. We hypothesized that the lower cardiac mortality associated with the more recent period under full follow-up would be attenuated when follow-up opportunity was standardized to 36 months. To separate the influence of the follow-up window from exclusion of the most recent diagnoses, we also repeated the full-follow-up analysis in patients diagnosed through 2018. A mutually exclusive surgery cohort served as a clinical benchmark for broader calendar-period patterns, and propensity score matching was used only as a descriptive sensitivity analysis rather than to estimate a causal treatment effect. The primary research question was whether cardiac mortality in the beam radiotherapy cohort was lower in 2015–2021 than in 2009–2014 under full follow-up and whether any association persisted in the common 36-month comparison of the 2015–2018 and 2009–2014 cohorts.

## Materials and methods

### Data source, study population, and cohort definition

We conducted a retrospective population-based cohort study using data from the Surveillance, Epidemiology, and End Results (SEER) Program of the National Cancer Institute. Records were obtained from the Incidence–SEER Research Data, 8 Registries, November 2023 Submission (1975–2021) dataset [10]. Patients with primary malignant tumors of the lung and bronchus diagnosed between January 1, 2009, and December 31, 2021, were identified using SEER*Stat software, version 8.4.4 (National Cancer Institute, Bethesda, MD, USA).

Eligibility required known age at diagnosis and sufficient survival and cause-of-death information for outcome classification. Records coded as having received chemotherapy were excluded; eligible records were therefore limited to those classified as “No/Unknown” in the SEER chemotherapy recode. Two mutually exclusive cohorts were defined using first-course treatment variables. The beam radiotherapy cohort included patients with recorded beam radiation and no primary-site surgery. The surgery cohort included patients with documented primary-site surgery and no recorded radiotherapy. Records with unknown, unspecified, or ambiguous surgical status were excluded.

After application of the eligibility and treatment-selection criteria, the original unmatched cohort included 56,241 patients: 28,246 in the beam radiotherapy cohort and 27,995 in the surgery cohort.

### Exposure, outcome, and covariates

For descriptive comparisons between treatment groups, the variable of interest was treatment cohort, classified as beam radiotherapy or primary-site surgery. For temporal analyses within each cohort, the variable of interest was diagnosis period. Under full follow-up, patients diagnosed in 2015–2021 were compared with those diagnosed in 2009–2014.

Two additional analyses used patients diagnosed through 2018. A restricted full-follow-up analysis compared patients diagnosed in 2015–2018 with those diagnosed in 2009–2014 while retaining all observed follow-up. The same diagnosis-period groups were then compared within a fixed 36-month window, with follow-up for every patient administratively censored at 36 months after diagnosis.

Diagnosis period was treated as an era marker rather than as an indicator of a specific radiotherapy technique. SEER does not provide information on treatment-planning technique, prescribed radiation dose, fractionation, treatment fields, or cardiac dosimetry.

The primary endpoint was cardiac death, defined using the “Diseases of Heart” category of the SEER Cause of Death Recode [11]. Death from lung and bronchus cancer was treated as the competing event. Deaths from other causes were censored at the recorded survival time, and patients who remained alive were censored at the end of follow-up.

Prespecified covariates were age group, sex, race, summary stage, histological subtype, and primary tumor site. Histological subtype was categorized using morphology codes from the International Classification of Diseases for Oncology, Third Edition (ICD-O-3), according to the framework described by Lewis et al. [12]. Categories comprised NSCLC, adenocarcinoma/adenosquamous; NSCLC, squamous cell carcinoma; NSCLC, large-cell carcinoma; SCLC; and carcinoma, NOS/other. Treatment cohort or diagnosis period was included as appropriate for the analysis being performed.

### Statistical analysis

Baseline characteristics were summarized as counts and percentages. Categorical variables were compared using Pearson’s chi-square test; Yates’ continuity correction was applied to 2 × 2 contingency tables. p values in the baseline tables were considered descriptive. Cumulative incidence functions were used to estimate cardiac mortality in the presence of competing death from lung and bronchus cancer, and differences between groups were assessed using Gray’s test [13]. The prespecified event-coding and censoring scheme described above was applied throughout the analyses. Survival time was expressed in months; records with zero recorded survival months were assigned a nominal survival time of 0.5 months to allow inclusion in the time-to-event analyses.

Fine–Gray subdistribution hazards regression was used for multivariable competing-risk analyses [14]. Estimates were reported as subdistribution hazard ratios (sHRs) with 95 % confidence intervals (CIs), consistent with recommended reporting practice [15]. Adjusted temporal models included diagnosis period, age group, sex, race, summary stage, histological subtype, and primary tumor site, with 2009–2014 as the reference diagnosis period.

In the full-follow-up analysis, patients diagnosed in 2015–2021 were compared with those diagnosed in 2009–2014. The diagnosis-restricted full-follow-up analysis included patients diagnosed through 2018 and compared 2015–2018 with 2009–2014 while retaining all observed follow-up. The fixed-window analysis used that same 2009–2018 cohort but administratively censored follow-up at 36 months. All three models were evaluated separately in the beam radiotherapy and surgery cohorts.

Propensity score matching was performed as a descriptive sensitivity analysis of the beam radiotherapy and surgery cohorts. Propensity scores for membership in the beam radiotherapy cohort were estimated using age group, sex, race, summary stage, histological subtype, and primary tumor site. Patients were matched 1:1 by nearest-neighbor matching without replacement, using a caliper width of 0.2 of the standard deviation of the logit of the estimated propensity score [16]. Diagnosis period was not included in the propensity score model because calendar-period associations were evaluated separately in the prespecified temporal analyses. Covariate balance before and after matching was assessed using standardized mean differences (SMDs), with an absolute SMD below 0.10 defined as the prespecified balance target [17]. The cumulative incidence framework was also applied to the matched cohort. Because matching did not address unmeasured confounding and residual imbalance remained in several measured variables, the matched comparison was not interpreted as a causal estimate of treatment effect.

To evaluate whether the diagnosis-period association depended on the observation window, we estimated period sHRs across fixed administrative windows of 12, 24, 36, 48, and 60 months; for each window we restricted eligibility to diagnoses with the corresponding potential follow-up and censored all patients at the window length. Because the eligible populations differed across window lengths, these estimates were interpreted as an exploratory pattern rather than as repeated estimates from an identical cohort. We tested the proportional-subdistribution-hazards assumption for diagnosis period by adding a period-by-time interaction to the full-follow-up radiotherapy model.

As sensitivity analyses, we repeated all period models treating all noncardiac deaths as competing events (the conventional Fine–Gray specification) and using cause-specific Cox regression with cardiac death as the event and all other deaths censored. We report reverse Kaplan–Meier median follow-up, person-time, and event counts by diagnosis period. The vital-status cutoff corresponded to the November 2023 SEER submission (follow-up through 2021); diagnoses through 2018 therefore had at least 36 months of potential follow-up. Records with zero recorded survival months (1,644 of 28,246 radiotherapy records, 5.8 %) were assigned a nominal survival time of 0.5 months; excluding them yielded essentially identical estimates (full-follow-up sHR 0.770; 36-month sHR 1.013).

All statistical tests were two-sided, and p<0.05 was considered statistically significant. Statistical analyses were performed using R version 4.6.0 (R Foundation for Statistical Computing, Vienna, Austria). Cumulative incidence functions, Gray’s tests, and Fine–Gray regression models were implemented using the cmprsk package, version 2.2.12. Propensity score matching was performed using MatchIt, version 4.7.2. Baseline tables and standardized mean differences were generated using tableone, version 0.13.2, and covariate balance was assessed using cobalt, version 4.6.3. Figures were created using ggplot2, version 4.0.3.

Ethics approval and informed consent: Not applicable. This study used de-identified data obtained from the Surveillance, Epidemiology, and End Results Program under the SEER data-use agreement and involved no direct contact with study participants.

## Results

### Cohort selection, baseline characteristics, and propensity score matching

**Figure 1:**
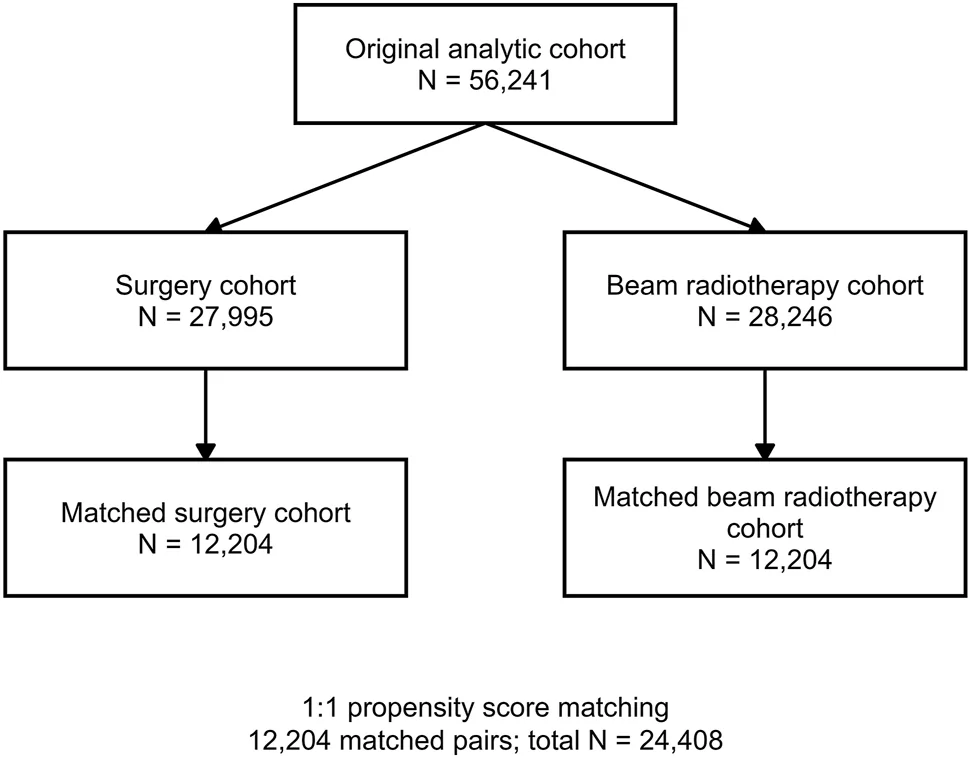
Study cohort composition and propensity score matching. The original unmatched cohort included 56,241 patients: 27,995 in the surgery cohort and 28,246 in the beam radiotherapy cohort. After 1:1 propensity score matching, 12,204 patients remained in each treatment cohort, resulting in a total matched cohort of 24,408 patients.

The original unmatched cohort included 56,241 patients, comprising 28,246 in the beam radiotherapy cohort and 27,995 in the surgery cohort (Figure 1). Compared with the surgery cohort, the beam radiotherapy cohort included a higher proportion of older patients, men, patients with distant-stage disease, and patients with small-cell lung cancer (Table 1).

**Table 1:** Baseline characteristics of the original unmatched cohort by treatment cohort.

Characteristic	Level	Surgery	Beam radiotherapy	p-Value	SMD
n		27,995	28,246		
Age group	15–59	4,718 (16.9)	2,660 (9.4)	<0.001	0.512
	60–69	9,562 (34.2)	6,779 (24.0)		
	70–79	10,516 (37.6)	10,294 (36.4)		
	≥80	3,199 (11.4)	8,513 (30.1)		
Sex	Female	15,938 (56.9)	13,610 (48.2)	<0.001	0.176
	Male	12,057 (43.1)	14,636 (51.8)		
Race	White	23,205 (82.9)	23,144 (81.9)	<0.001	0.084
	Black	2,043 (7.3)	2,633 (9.3)		
	Other	2,660 (9.5)	2,425 (8.6)		
	Unknown	87 (0.3)	44 (0.2)		
Diagnosis period	2009–2014	12,629 (45.1)	11,124 (39.4)	<0.001	0.116
	2015–2021	15,366 (54.9)	17,122 (60.6)		
Summary stage	Localized	19,239 (68.7)	12,716 (45.0)	<0.001	1.043
	Regional	7,530 (26.9)	3,335 (11.8)		
	Distant	1,105 (3.9)	11,948 (42.3)		
	Unknown	121 (0.4)	247 (0.9)		
Histology	NSCLC, adenocarcinoma/adenosquamous	16,043 (57.3)	13,224 (46.8)	<0.001	0.563
	NSCLC, squamous	5,228 (18.7)	7,390 (26.2)		
	NSCLC, large-cell	834 (3.0)	388 (1.4)		
	SCLC	438 (1.6)	3,929 (13.9)		
	Carcinoma, NOS/other	5,452 (19.5)	3,315 (11.7)		
Primary site	Upper lobe	15,657 (55.9)	15,714 (55.6)	<0.001	0.175
	Lower lobe	9,530 (34.0)	8,190 (29.0)		
	Other lung site	2,808 (10.0)	4,342 (15.4)		

Data are n (%). p values were calculated using Pearson’s chi-square test; Yates’ continuity correction was applied to 2 × 2 contingency tables. p values and standardized mean differences are descriptive. SMD, standardized mean difference; NSCLC, non-small-cell lung cancer; SCLC, small-cell lung cancer; NOS, not otherwise specified.

The original cohort included 2,078 cardiac deaths and 19,491 deaths from lung and bronchus cancer. Of these, 1,153 cardiac deaths and 14,462 lung and bronchus cancer deaths occurred in the beam radiotherapy cohort, whereas 925 cardiac deaths and 5,029 lung and bronchus cancer deaths occurred in the surgery cohort (Table 2).

**Table 2:** Key analytic cohorts and event counts.

Cohort	n	Cardiac deaths	Deaths from lung and bronchus cancer
Original cohort	56,241	2,078	19,491
Surgery cohort	27,995	925	5,029
Beam radiotherapy cohort	28,246	1,153	14,462
Propensity score-matched cohort	24,408	1,145	7,429

Cardiac death was the event of interest, and death from lung and bronchus cancer was the prespecified competing event. The propensity score-matched row represents the combined matched cohort of 12,204 patients in each treatment cohort.

After 1:1 propensity score matching, 24,408 patients remained, with 12,204 in each treatment cohort. The matched cohort included 1,145 cardiac deaths and 7,429 deaths from lung and bronchus cancer. Matching substantially improved balance in age and sex. Race remained within the prespecified balance threshold before and after matching, whereas residual imbalance persisted for histological subtype, summary stage, and primary tumor site (Table S1 and Figure S1). Diagnosis period also remained imbalanced because it was not included in the propensity score model. The matched cohort was therefore used only for a descriptive sensitivity analysis.

### Cardiac mortality by treatment cohort

**Figure 2:**
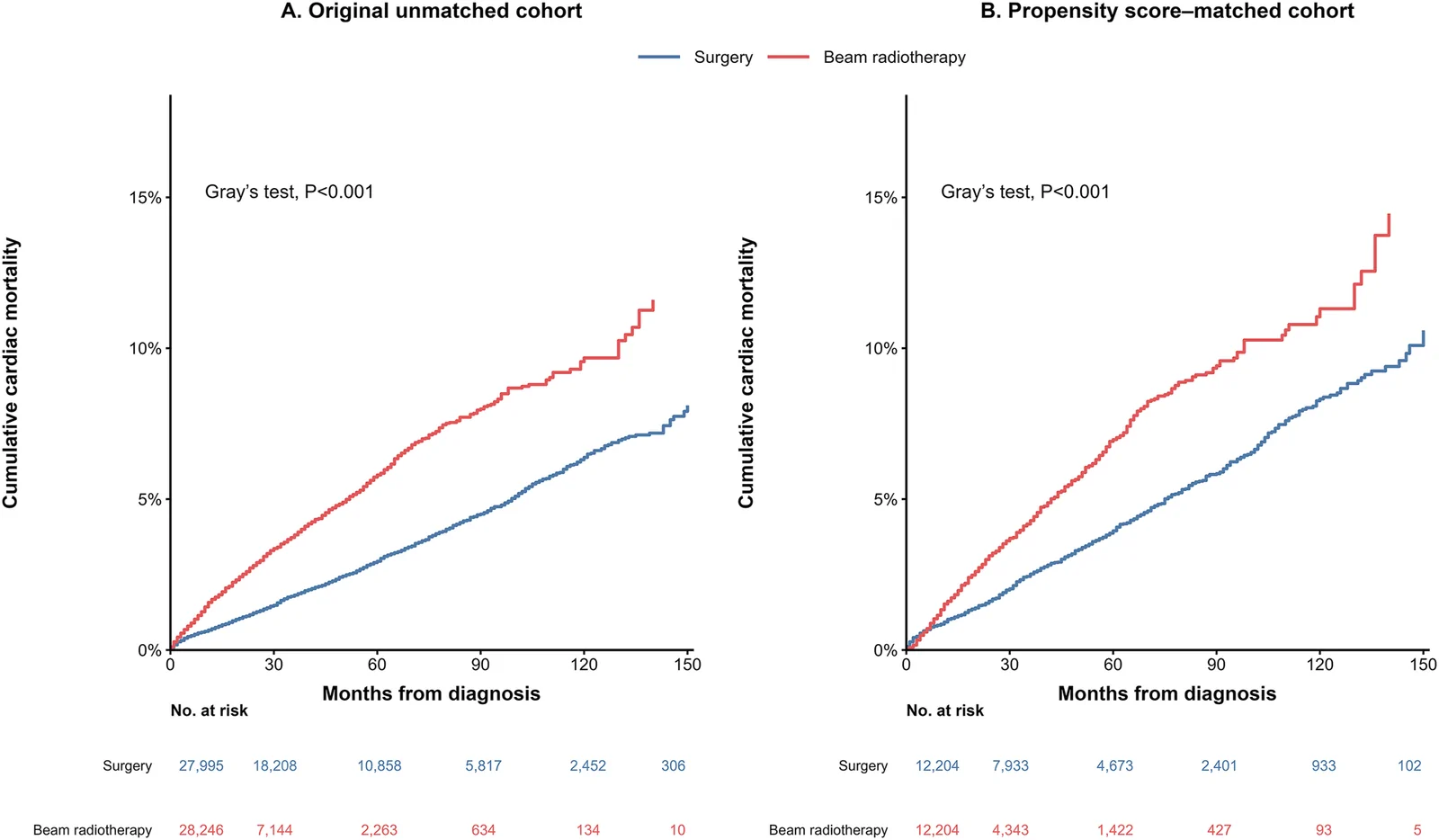
Cumulative incidence of cardiac death by treatment cohort. (A) Original unmatched cohort. (B) Propensity score-matched cohort. Death from lung and bronchus cancer was the competing event; Gray’s test yielded p<0.001 in both panels. Numbers below each panel show those at risk. Comparisons are descriptive because treatment-selection differences and residual imbalance remained.

The cumulative incidence of cardiac death was higher in the beam radiotherapy cohort than in the surgery cohort in both the original unmatched sample and the propensity score-matched sample (Gray’s test, p<0.001 for both comparisons; Figure 2A and B). Because substantial treatment-selection differences remained, including residual imbalance after matching, these comparisons were considered descriptive and were not interpreted as causal effects of treatment modality.

### Full-follow-up temporal trends

**Figure 3:**
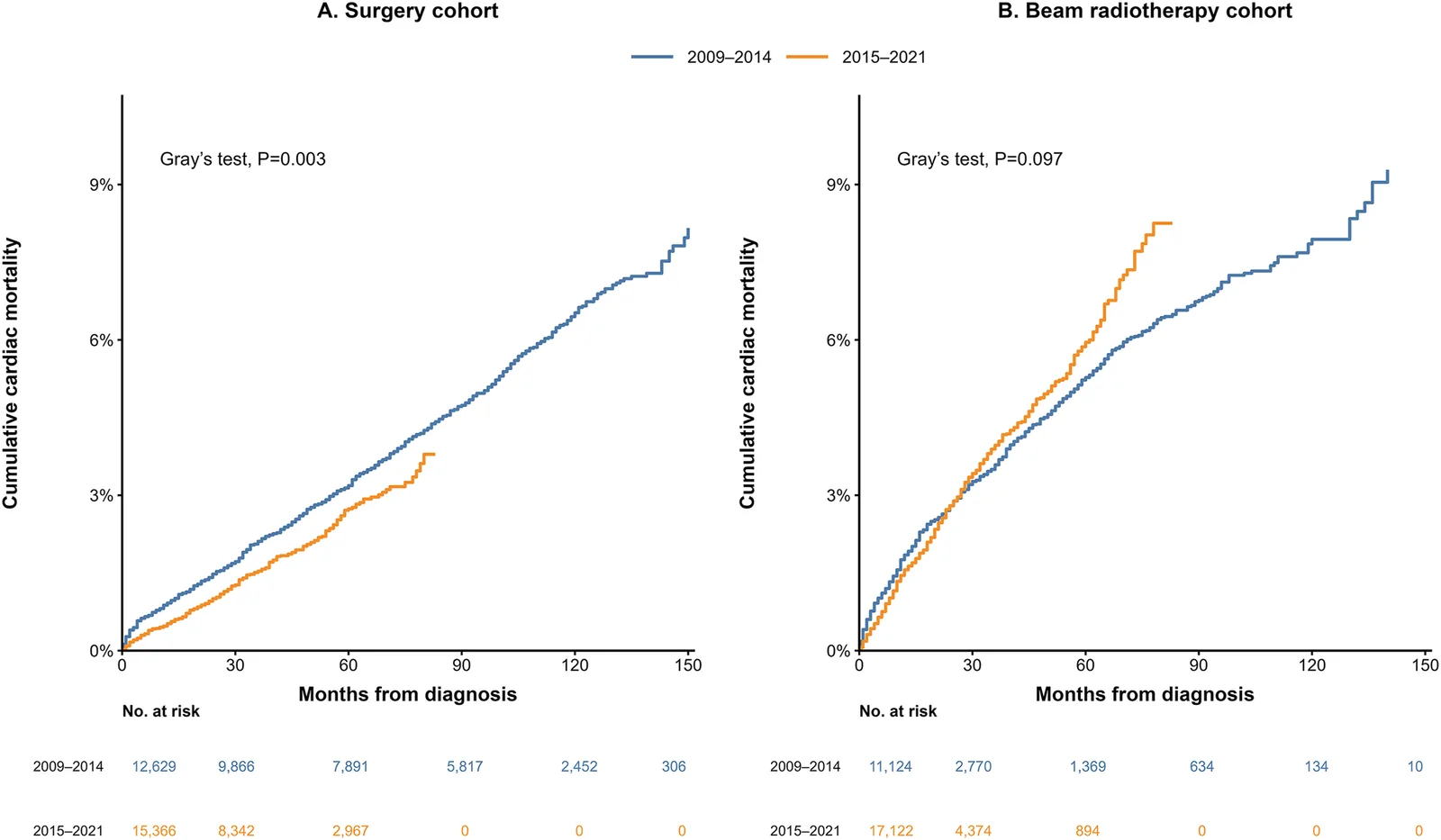
Full-follow-up cumulative incidence of cardiac death by diagnosis period. (A) Surgery cohort. (B) Beam radiotherapy cohort. Curves compare 2009–2014 with 2015–2021; death from lung and bronchus cancer was the competing event. Gray’s test yielded p=0.003 and p=0.097, respectively. Numbers below each panel show those at risk. The comparisons are descriptive because potential follow-up differed by diagnosis period.

Under full follow-up, the cumulative incidence of cardiac death differed between diagnosis periods in the surgery cohort (Gray’s test, p=0.003; Figure 3A). No statistically significant difference was observed in the beam radiotherapy cohort (Gray’s test, p=0.097; Figure 3B). Because patients diagnosed in 2015–2021 had less potential follow-up than those diagnosed in 2009–2014, these unadjusted comparisons were considered descriptive.

### Fixed 36-month temporal analysis

**Figure 4:**
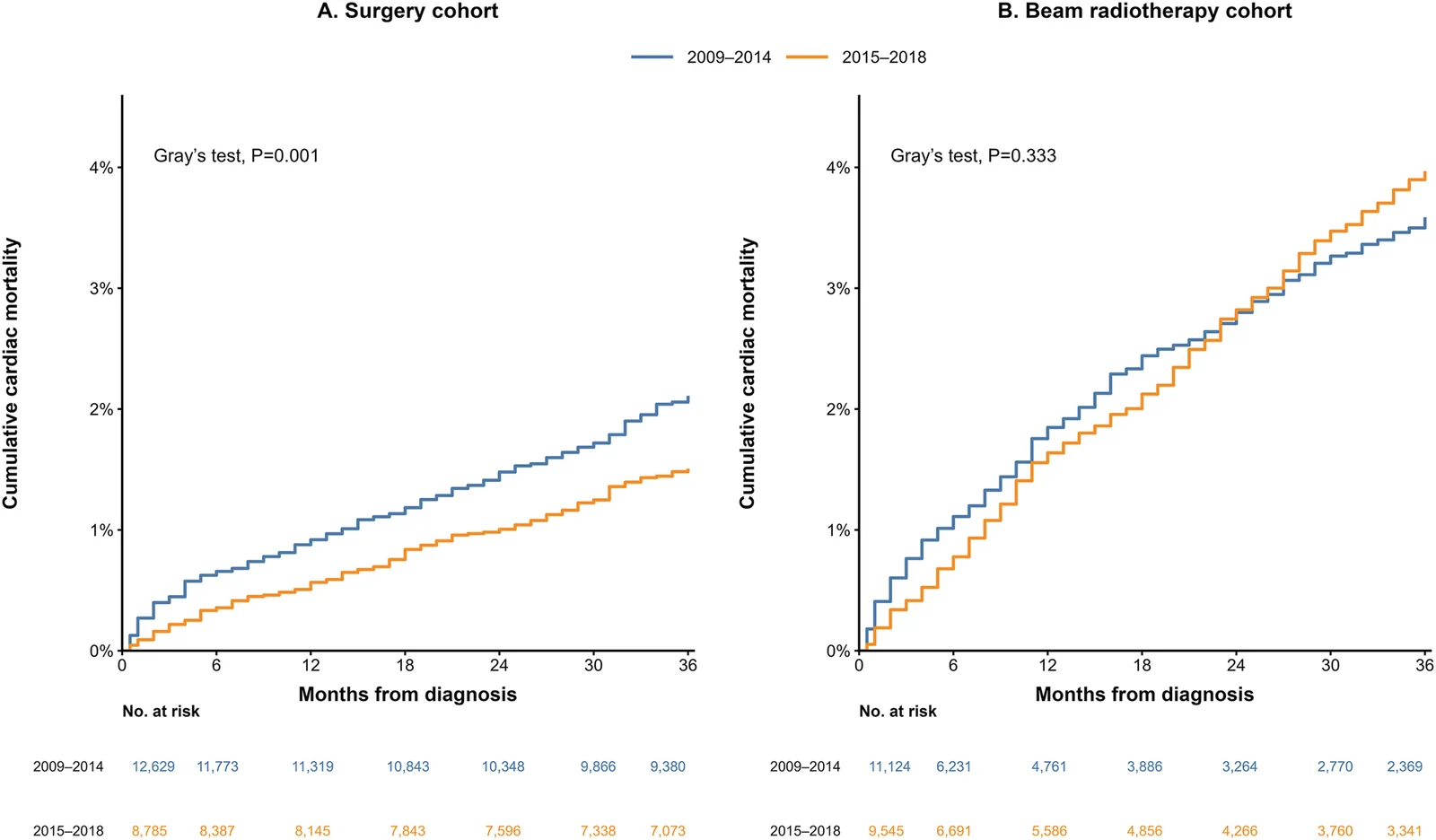
Cumulative incidence of cardiac death by diagnosis period within a fixed 36-month window. (A) Surgery cohort. (B) Beam radiotherapy cohort. Patients diagnosed in 2009–2014 were compared with those diagnosed in 2015–2018, and follow-up for patients in both diagnosis-period groups was administratively censored at 36 months after diagnosis. Death from lung and bronchus cancer was treated as the prespecified competing event. Gray’s test yielded p=0.001 in the surgery cohort and p=0.333 in the beam radiotherapy cohort. Numbers below each panel show the numbers at risk.

Within the fixed 36-month window, the cumulative incidence of cardiac death differed between diagnosis periods in the surgery cohort (Gray’s test, p=0.001; Figure 4A). No statistically significant difference was observed in the beam radiotherapy cohort (Gray’s test, p=0.333; Figure 4B).

In the beam radiotherapy cohort, cardiac death within 36 months occurred in 346 of 11,124 patients (3.11 %) diagnosed in 2009–2014 and in 331 of 9,545 patients (3.47 %) diagnosed in 2015–2018. These crude event proportions were reported descriptively and were not substitutes for the competing-risk cumulative incidence estimates.

### Adjusted Fine–Gray diagnosis-period associations

**Figure 5:**
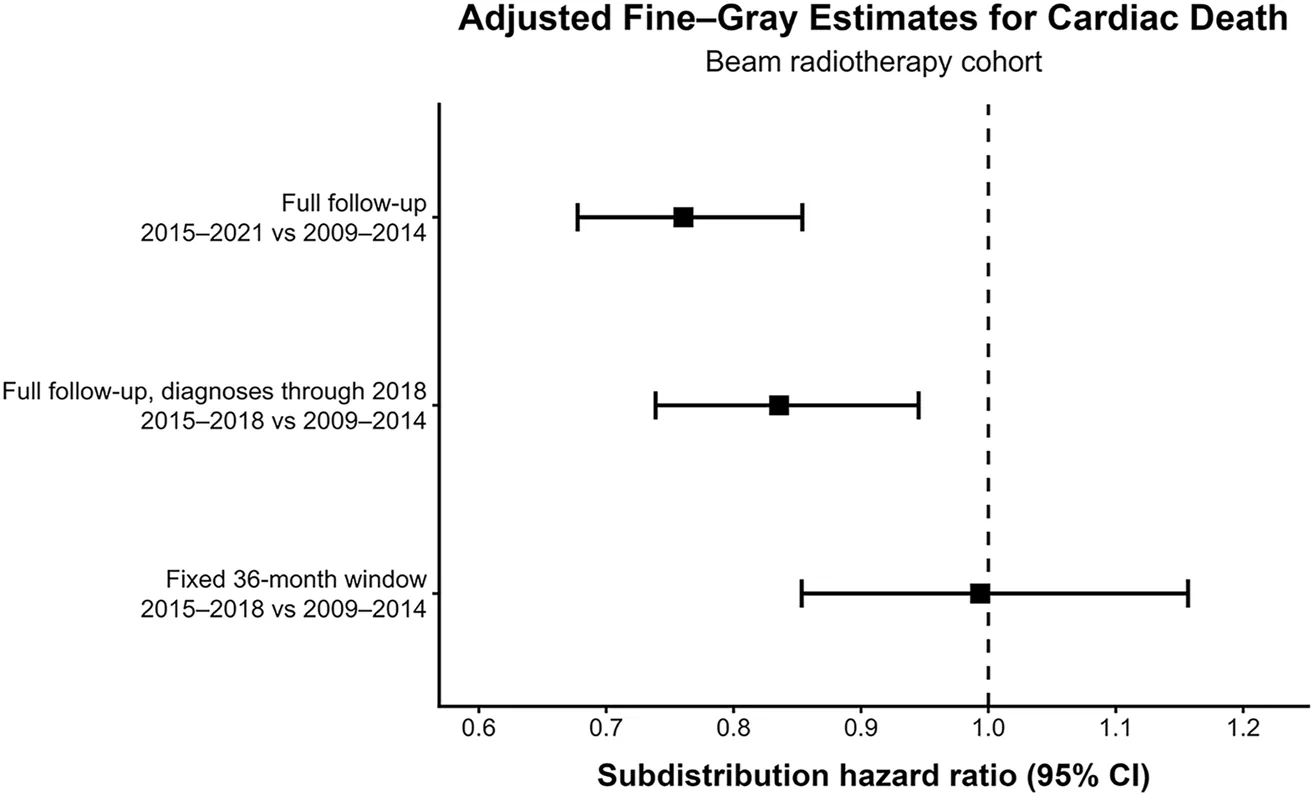
Adjusted Fine–Gray estimates of diagnosis-period associations with cardiac death in the beam radiotherapy cohort. Models compared 2015–2021 with 2009–2014 under full follow-up and 2015–2018 with 2009–2014 in the diagnosis-restricted full-follow-up and fixed 36-month analyses. The latter two models used the same 2009–2018 cohort; only the fixed-window model censored follow-up at 36 months. Models adjusted for age group, sex, race, summary stage, histological subtype, and primary tumor site. Squares show sHRs, horizontal lines show 95 % CIs, and the dashed line indicates an sHR of 1.0.

In the beam radiotherapy cohort, diagnosis in 2015–2021 was associated with a lower subdistribution hazard of cardiac death than diagnosis in 2009–2014 under full follow-up (sHR 0.761; 95 % CI 0.678–0.854; p<0.001). Restricting to diagnoses through 2018 while retaining all observed follow-up moved the estimate toward the null (sHR 0.836; 95 % CI 0.739–0.945; p=0.004), and administrative censoring of that same 2009–2018 cohort at 36 months removed the association (sHR 0.994; 95 % CI 0.853–1.157; p=0.930) (Table 3; Figure 5). The radiotherapy cohort’s full-follow-up median observation was 108 months for 2009–2014 vs. 38 months for 2015–2021 (Table 5), and the period effect was non-proportional over follow-up time (period-by-time interaction p=0.003), indicating that the single full-follow-up sHR is a time-averaged quantity weighted toward the longer-followed earlier cohort.

**Table 3:** Adjusted Fine–Gray estimates of diagnosis-period associations by cohort and follow-up framework.

Cohort	Follow-up framework	Comparison	sHR (95 % CI)	p-Value
Beam radiotherapy	Full follow-up	2015–2021 vs. 2009–2014	0.761 (0.678–0.854)	<0.001
Beam radiotherapy	Full follow-up, diagnoses through 2018	2015–2018 vs. 2009–2014	0.836 (0.739–0.945)	0.004
Beam radiotherapy	Fixed 36-month window	2015–2018 vs. 2009–2014	0.994 (0.853–1.157)	0.930
Surgery	Full follow-up	2015–2021 vs. 2009–2014	0.721 (0.622–0.837)	<0.001
Surgery	Full follow-up, diagnoses through 2018	2015–2018 vs. 2009–2014	0.734 (0.626–0.859)	<0.001
Surgery	Fixed 36-month window	2015–2018 vs. 2009–2014	0.770 (0.622–0.954)	0.017

Cardiac death was the event of interest, and death from lung and bronchus cancer was the prespecified competing event. Models were adjusted for age group, sex, race, summary stage, histological subtype, and primary tumor site. The reference diagnosis period was 2009–2014. The full-follow-up analyses restricted to diagnoses through 2018 used the same 2009–2018 cohort within each treatment group as the fixed 36-month analyses but retained all observed follow-up. sHR, subdistribution hazard ratio; CI, confidence interval.

In the surgery cohort, the recent-period estimate was lower under full follow-up (sHR 0.721; 95 % CI 0.622–0.837; p<0.001) and – when run through the identical restricted-full and fixed-window sequence – remained significantly below 1.0 (restricted-full sHR 0.734; 95 % CI 0.626–0.859; p<0.001; 36-month sHR 0.770; 95 % CI 0.622–0.954; p=0.017) (Table 3).

### Window-length surface and sensitivity to analytic choices

**Figure 6:**
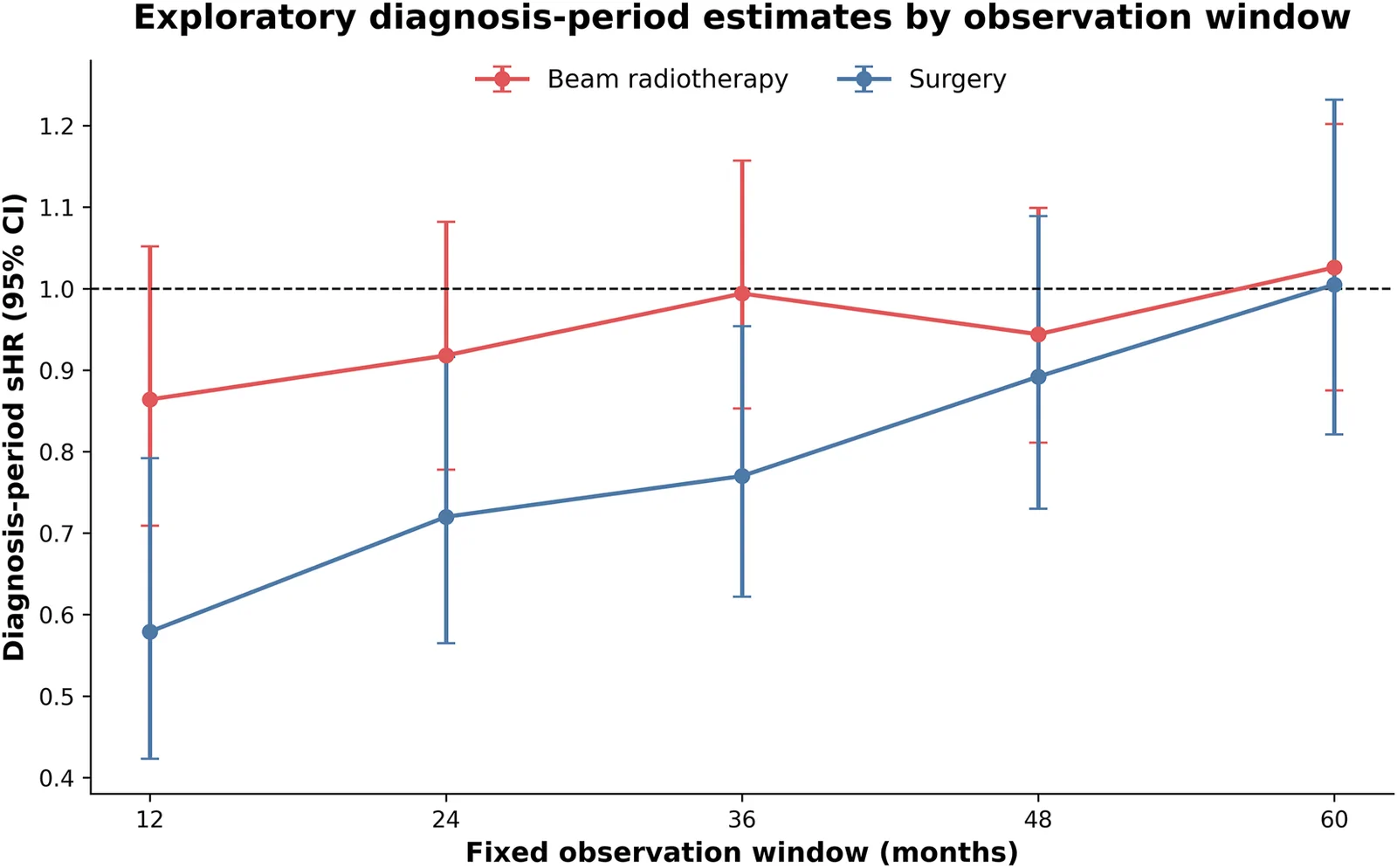
Diagnosis-period subdistribution hazard ratios across fixed observation windows. Points show adjusted sHRs and vertical lines show 95 % CIs for the beam radiotherapy and surgery cohorts. The dashed horizontal line indicates an sHR of 1.0. Eligibility was restricted separately for each window to diagnosis years with the corresponding potential follow-up; the populations therefore differed across window lengths.

Across fixed windows of 12–60 months, the radiotherapy period estimate was non-significant at every window (sHR 0.864, 0.918, 0.994, 0.944, and 1.026 at 12, 24, 36, 48, and 60 months; all p≥0.14), despite 409–696 cardiac events per window, whereas the surgery estimate was significant at 12–36 months (sHR 0.579, 0.720, and 0.770; p<0.02) and attenuated only at 48–60 months (Table 4; Figure 6).

**Table 4:** Window-length sensitivity surface (diagnosis-period sHR by fixed observation window).

Window, months	Radiotherapy sHR (95 % CI)	Radiotherapy p-Value	Radiotherapy cardiac deaths	Surgery sHR (95 % CI)	Surgery p-Value	Surgery cardiac deaths
12	0.864 (0.709–1.052)	0.140	409	0.579 (0.423–0.792)	<0.001	176
24	0.918 (0.778–1.082)	0.310	587	0.720 (0.565–0.916)	0.008	287
36	0.994 (0.853–1.157)	0.930	677	0.770 (0.622–0.954)	0.017	381
48	0.944 (0.811–1.099)	0.460	696	0.892 (0.730–1.089)	0.260	454
60	1.026 (0.875–1.202)	0.750	689	1.005 (0.821–1.232)	0.960	497

For each window, eligibility was restricted to diagnosis years with the corresponding potential follow-up, and all patients were administratively censored at the specified window. The eligible populations therefore differed across windows; the estimates should be interpreted as an exploratory surface rather than repeated estimates from an identical cohort. Models were adjusted for age group, sex, race, summary stage, histological subtype, and primary tumor site. sHR, subdistribution hazard ratio; CI, confidence interval.

The radiotherapy attenuation was unchanged when all noncardiac deaths were modeled as competing events (36-month sHR 1.000; 95 % CI 0.860–1.164; p=0.996) and under cause-specific Cox regression (36-month HR 0.898; 95 % CI 0.770–1.046; p=0.167); the surgery estimate remained significant at 36 months under both alternatives (Tables S3 and S4). As a horizon-anchored estimand, the 36-month cumulative incidence of cardiac death in the radiotherapy cohort did not differ between periods (2015–2018, 3.97 %; 2009–2014, 3.59 %; difference +0.38 percentage points; 95 % CI −0.18 to +0.94; Table S6). In a localized-stage subgroup, the recent-period association was significant under full follow-up (sHR 0.848; 95 % CI 0.735–0.977; p=0.023) but null within the 36-month window (sHR 1.125; 95 % CI 0.920–1.376; p=0.250) (Table S5).

## Discussion

In this competing-risks analysis, the apparent association between more recent diagnosis and lower cardiac mortality after lung radiotherapy was not robust to the follow-up horizon. Although the full-follow-up subdistribution hazard ratio was 0.761, the association was absent at every equalized observation window from 12 to 60 months, remained null under both alternative competing-risk coding and cause-specific regression, and reflected a formally non-proportional, time-varying period effect superimposed on a large follow-up imbalance (median 108 vs. 38 months). A parsimonious interpretation is that the full-follow-up estimate is strongly influenced by the longer-followed earlier cohort rather than demonstrating a reduction in cardiac mortality among more recently treated radiotherapy patients.

The surgery cohort, analyzed identically, behaved differently and was informative as a clinical benchmark. Its recent-period estimate remained significantly below 1.0 through a 36-month window and attenuated only at longer windows, demonstrating that matched-window analysis does not mechanically eliminate every calendar-period signal. The contrast between cohorts – radiotherapy null at all windows, surgery significant at the same 12–36-month windows – indicates that a recent-period reduction was detectable in at least one comparison cohort but was absent in the radiotherapy cohort once follow-up was equalized. This differential, rather than a simple “follow-up artifact,” is the central finding: a calendar-period reduction may reflect broader cardiovascular-care advances, secular changes in population cardiac mortality, or stage migration, but registry data provide no evidence that any reduction was driven by changes in radiotherapy practice.

These observations re-appraise prior registry reports of declining cardiac mortality in more recent radiotherapy eras [9]. Such reports are vulnerable because recently diagnosed patients have shorter potential follow-up for a late-onset endpoint; when the same cohort is examined at comparable follow-up, the recent-era radiotherapy advantage does not persist. Because our cohort excludes chemotherapy-recorded patients, we do not re-analyze the specific locally advanced population of [9]; rather, we demonstrate the general fragility of calendar-period radiotherapy claims derived from registries that lack treatment technique and cardiac dosimetry. Established methodological alternatives include period analysis [18], fixed-horizon cumulative-incidence or restricted-mean comparisons with explicit estimands, age-period-cohort decomposition, and, for treatment-technique questions, target-trial emulation [19]. We report the 36-month cumulative-incidence difference with its confidence interval as a horizon-anchored estimand and emphasize that the full-follow-up and fixed-window sHRs are different estimands, so their comparison reflects a time-varying association rather than a single effect observed with more or less precision.

Prior dose-based studies addressed a different question, relating measured whole-heart or cardiac-substructure exposure and baseline cardiac risk to cardiac events and survival [6], [7], [8], building on foundational dose-response evidence for radiation-related ischemic heart disease [20]. SEER contains no cardiac dosimetry, radiotherapy technique, dose, or fractionation; diagnosis period is therefore an era marker that may capture, among other changes, the diffusion of intensity-modulated radiotherapy [21] and stereotactic body radiotherapy across 2009–2021. The radiotherapy cohort is clinically heterogeneous – spanning stereotactic radiotherapy for medically inoperable early-stage disease, definitive radiotherapy alone, and palliative thoracic radiotherapy for advanced or small-cell disease – and the exclusion of chemotherapy-recorded patients removes the concurrent-chemoradiation population in which much of the cardiac-dose literature was generated. Results should not be extrapolated to that setting.

Competing death from lung and bronchus cancer is central to interpretation of cardiac mortality in this population. In the adjusted radiotherapy model, regional and distant disease were associated with lower subdistribution hazards of cardiac death than localized disease (Table S2). This does not imply lower underlying cardiovascular risk in patients with more advanced cancer. Earlier lung cancer death prevents a later cardiac death from being observed and reduces the cumulative occurrence of the cardiac endpoint within the prespecified competing-risk framework [14]. Stage may also capture differences in expected survival, performance status, and treatment intent that cannot be fully characterized in SEER. The stage estimates are therefore best understood in the context of competing mortality, not as a protective effect of advanced disease or its treatment.

The fixed 36-month analysis improved comparability of follow-up opportunity but covered only early to intermediate outcomes. Radiation-related cardiovascular disease may have a longer clinical latency, with some manifestations occurring beyond 3 years [22]. Cardiac death was also the only cardiovascular endpoint available; nonfatal and subclinical injury could not be assessed. The near-null estimate within 36 months is thus limited to cardiac mortality observed during that interval and does not exclude later morbidity or mortality. Current guidance continues to recommend baseline cardiovascular risk assessment and risk-adapted surveillance [23], while thoracic radiotherapy guidance emphasizes minimizing cardiac exposure and tailoring preventive strategies to baseline risk [24]. Nor does the fixed-window result imply that contemporary radiotherapy failed to reduce cardiac exposure or nonfatal injury. This registry analysis cannot define surveillance intervals, imaging protocols, or cardioprotective interventions. Its main practical implication is methodological: temporal comparisons should use comparable follow-up windows and account for competing lung cancer death before differences are linked to changes in treatment practice.

The study combined a large population-based cohort with competing-risk methods suited to a population with substantial lung cancer mortality. A particular strength was the evaluation of the same 2009–2018 radiotherapy cohort with and without administrative censoring at 36 months. Treatment-cohort and matched comparisons were kept descriptive, and the three temporal models were used to examine the stability of the diagnosis-period estimate rather than to establish a treatment effect. Even so, residual confounding remains. Propensity score matching can address only recorded covariates included in the model [25]. SEER lacks pre-existing cardiovascular disease, smoking history, cardiac function, medication use, frailty, pulmonary function, performance status, operability, and treatment intent. Calendar-period estimates may also reflect unmeasured changes in case mix, diagnostic and staging practices, supportive care, and treatment selection. Tumor metabolic and immune heterogeneity, which may influence lung cancer progression and treatment response, was likewise unavailable and could have affected the competing risk of lung cancer death [26]. Multivariable adjustment and matching could not isolate radiotherapy practice from these other era-related changes.

Registry coding imposed additional limitations. Cardiac death was identified using the broad SEER “Diseases of Heart” category [11], which does not distinguish specific cardiovascular conditions or determine whether a death was related to radiotherapy. Death-certificate classification may also be inaccurate [27]. The SEER chemotherapy recode combines patients with no documented chemotherapy and those with unknown treatment status. Because patients recorded as receiving chemotherapy were excluded, this was not a confirmed chemotherapy-naive cohort. Incomplete treatment ascertainment may have introduced selection and residual confounding and may limit generalizability [28]. The primary analyses treated lung and bronchus cancer death as the prespecified competing event and censored deaths from other causes; however, alternative coding that treated all noncardiac deaths as competing events yielded materially similar results, as did cause-specific Cox regression.

Two further considerations bear on interpretation. First, changes in lung-cancer mortality after 2015, including those associated with the introduction of immune checkpoint inhibitors, may alter the incidence of the competing event and move a subdistribution hazard ratio independently of any change in cardiac risk [1]. We therefore report cause-specific hazards, which are not mechanically tied to the competing event and showed the same attenuation, together with per-period competing-event counts (Table 5). Second, completeness of the SEER chemotherapy recode may vary by calendar period, so the exclusion of chemotherapy-recorded patients is itself potentially period-correlated; the all-competing-risk and localized-stage sensitivity analyses bound but do not eliminate this concern.

**Table 5:** Follow-up duration, person-time, and event counts by cohort, diagnosis period, and follow-up framework.

Cohort	Follow-up framework	Diagnosis period	n	Person-years	Cardiac deaths	Lung cancer deaths	Other deaths	Alive or censored	Median follow-up, months (95 % CI)
Beam radiotherapy	Full follow-up	2009–2014	11,124	20,321	582	7,561	2,411	570	108 (105–110)
Beam radiotherapy	Full follow-up	2015–2021	17,122	27,403	571	6,901	2,677	6,973	38 (37–38)
Beam radiotherapy	Full follow-up, diagnoses through 2018	2009–2014	11,124	20,321	582	7,561	2,411	570	108 (105–110)
Beam radiotherapy	Full follow-up, diagnoses through 2018	2015–2018	9,545	19,925	440	4,843	1,891	2,371	56 (55–57)
Beam radiotherapy	Fixed 36-month window	2009–2014	11,124	13,216	346	6,833	1,596	2,349	36 (36–36)
Beam radiotherapy	Fixed 36-month window	2015–2018	9,545	15,425	331	4,388	1,453	3,373	36 (36–36)
Surgery	Full follow-up	2009–2014	12,629	80,284	666	3,488	3,022	5,453	117 (116–118)
Surgery	Full follow-up	2015–2021	15,366	44,945	259	1,541	1,104	12,462	40 (40–41)
Surgery	Full follow-up, diagnoses through 2018	2009–2014	12,629	80,284	666	3,488	3,022	5,453	117 (116–118)
Surgery	Full follow-up, diagnoses through 2018	2015–2018	8,785	35,902	208	1,275	892	6,410	58 (57–59)
Surgery	Fixed 36-month window	2009–2014	12,629	32,423	254	1,924	1,069	9,382	36 (36–36)
Surgery	Fixed 36-month window	2015–2018	8,785	23,537	127	938	502	7,218	36 (36–36)

Person-time was calculated using observed follow-up for full-follow-up analyses and follow-up truncated at 36 months for the fixed-window analysis. Median follow-up and 95 % confidence intervals were estimated using the reverse Kaplan–Meier method. The four outcome categories sum to the cohort total in every row.

## Conclusions

Among patients with lung cancer treated with beam radiotherapy, a recent-era association with lower cardiac mortality under full follow-up did not persist at any comparable follow-up window, was robust to alternative competing-risk coding and cause-specific modeling, and reflected a non-proportional, time-varying association under markedly unequal follow-up. A recent-period reduction nonetheless persisted in the surgery comparison cohort through the 36-month matched-window analysis. These data indicate a population-level calendar-period pattern that cannot be attributed to changes in radiotherapy practice using registry information alone, and they show that temporal registry analyses should use comparable follow-up windows, model competing death appropriately, and verify proportionality before linking calendar-period change to treatment. Studies incorporating radiotherapy technique, cardiac dosimetry, baseline cardiovascular risk, and longer standardized follow-up – ideally framed as target-trial emulations – are required to determine whether contemporary radiotherapy has reduced cardiac mortality.

## Supplementary Material

Supplementary Material
