# Supplementary material for: Follow-up–dependent calendar-period associations with cardiac mortality after lung cancer radiotherapy: a competing-risks SEER analysis
Source: Open Med (Wars). 2026 Aug 25;21(1):20261509. doi: 10.1515/med-2026-1509 (PMC13576467; doi:10.1515/med-2026-1509)
Supplement: Supplementary file 1 [file j_med-2026-1509_suppl_001.docx]

Supplementary material

*Follow-up–dependent calendar-period associations with cardiac mortality after lung cancer radiotherapy: a competing-risks SEER analysis*

## Table S1. Baseline characteristics of the propensity score-matched cohort by treatment cohort.

| **Characteristic** | **Level** | **Surgery** | **Beam radiotherapy** | **P value** | **SMD** |
| --- | --- | --- | --- | --- | --- |
| N |  | 12,204 | 12,204 |  |  |
| Age group | 15–59 | 862 (7.1) | 1,111 (9.1) | <0.001 | 0.082 |
|  | 60–69 | 3,515 (28.8) | 3,276 (26.8) |  |  |
|  | 70–79 | 5,516 (45.2) | 5,447 (44.6) |  |  |
|  | ≥80 | 2,311 (18.9) | 2,370 (19.4) |  |  |
| Sex | Female | 6,011 (49.3) | 6,104 (50.0) | 0.239 | 0.015 |
|  | Male | 6,193 (50.7) | 6,100 (50.0) |  |  |
| Race | White | 10,230 (83.8) | 9,806 (80.4) | <0.001 | 0.092 |
|  | Black | 1,010 (8.3) | 1,254 (10.3) |  |  |
|  | Other | 947 (7.8) | 1,119 (9.2) |  |  |
|  | Unknown | 17 (0.1) | 25 (0.2) |  |  |
| Diagnosis period | 2009–2014 | 6,369 (52.2) | 4,234 (34.7) | <0.001 | 0.359 |
|  | 2015–2021 | 5,835 (47.8) | 7,970 (65.3) |  |  |
| Summary stage | Localized | 8,680 (71.1) | 8,070 (66.1) | <0.001 | 0.148 |
|  | Regional | 2,456 (20.1) | 2,533 (20.8) |  |  |
|  | Distant | 981 (8.0) | 1,434 (11.8) |  |  |
|  | Unknown | 87 (0.7) | 167 (1.4) |  |  |
| Histology | NSCLC, adenocarcinoma/adenosquamous | 6,858 (56.2) | 6,107 (50.0) | <0.001 | 0.225 |
|  | NSCLC, squamous | 4,158 (34.1) | 4,022 (33.0) |  |  |
|  | NSCLC, large-cell | 236 (1.9) | 292 (2.4) |  |  |
|  | SCLC | 408 (3.3) | 746 (6.1) |  |  |
|  | Carcinoma, NOS/other | 544 (4.5) | 1,037 (8.5) |  |  |
| Primary site | Upper lobe | 7,140 (58.5) | 6,936 (56.8) | <0.001 | 0.105 |
|  | Lower lobe | 4,035 (33.1) | 3,855 (31.6) |  |  |
|  | Other lung site | 1,029 (8.4) | 1,413 (11.6) |  |  |

*Data are n (%). P values were calculated using Pearson’s chi-square test; Yates’ continuity correction was applied to 2 × 2 contingency tables. P values and standardized mean differences are descriptive. The propensity score model included age group, sex, race, summary stage, histological subtype, and primary tumor site. Diagnosis period was reported descriptively but was not included in the propensity score model because calendar-period associations were evaluated separately. Residual imbalance remained in selected measured covariates; therefore, the matched cohort was used only for descriptive sensitivity analyses. An absolute SMD of 0.10 or greater indicates that the prespecified balance target was not met. SMD, standardized mean difference; NSCLC, non-small-cell lung cancer; SCLC, small-cell lung cancer; NOS, not otherwise specified.*

## Table S2. Adjusted Fine–Gray model for cardiac death in the beam radiotherapy cohort under full follow-up.

| **Covariate (reference)** | **sHR (95% CI)** | **P value** |
| --- | --- | --- |
| Diagnosis period: 2009–2014 | Reference |  |
| 2015–2021 | 0.76 (0.68–0.85) | <0.001 |
| Age group: 15–59 | Reference |  |
| 60–69 | 1.33 (0.95–1.86) | 0.10 |
| 70–79 | 2.05 (1.49–2.83) | <0.001 |
| ≥80 | 2.89 (2.10–3.98) | <0.001 |
| Sex: Female | Reference |  |
| Male | 1.55 (1.38–1.75) | <0.001 |
| Race: Black | Reference |  |
| White | 0.87 (0.71–1.07) | 0.19 |
| Other | 0.66 (0.49–0.89) | 0.007 |
| Unknown | 0.74 (0.10–5.43) | 0.77 |
| Histology: Carcinoma, NOS/other | Reference |  |
| NSCLC, adenocarcinoma/adenosquamous | 0.86 (0.75–1.00) | 0.048 |
| NSCLC, squamous | 1.10 (0.94–1.28) | 0.24 |
| NSCLC, large-cell | 0.14 (0.02–0.99) | 0.049 |
| SCLC | 0.64 (0.40–1.02) | 0.06 |
| Primary site: Upper lobe | Reference |  |
| Lower lobe | 1.03 (0.90–1.17) | 0.68 |
| Other lung site | 1.09 (0.90–1.32) | 0.37 |
| Summary stage: Localized | Reference |  |
| Regional | 0.75 (0.64–0.89) | <0.001 |
| Distant | 0.33 (0.28–0.39) | <0.001 |
| Unknown | 0.39 (0.17–0.86) | 0.02 |

*Subdistribution hazard ratios were estimated from a single multivariable Fine–Gray model with cardiac death as the event of interest and death from lung and bronchus cancer as the prespecified competing event. Deaths from other causes were censored according to the prespecified event-coding scheme. The model included diagnosis period, age group, sex, race, histological subtype, primary tumor site, and summary stage. The reference diagnosis period was 2009–2014. sHR, subdistribution hazard ratio; CI, confidence interval; NSCLC, non-small-cell lung cancer; SCLC, small-cell lung cancer; NOS, not otherwise specified.*

## Table S3. Fine–Gray sensitivity analyses treating all noncardiac deaths as competing events by cohort and follow-up framework.

| **Cohort** | **Follow-up framework** | **Comparison** | **N** | **Cardiac deaths** | **sHR (95% CI)** | **P value** |
| --- | --- | --- | --- | --- | --- | --- |
| Beam radiotherapy | Full follow-up | 2015–2021 vs 2009–2014 | 28,246 | 1,153 | 0.718 (0.640–0.806) | <0.001 |
| Beam radiotherapy | Full follow-up, diagnoses through 2018 | 2015–2018 vs 2009–2014 | 20,669 | 1,022 | 0.808 (0.714–0.914) | <0.001 |
| Beam radiotherapy | Fixed 36-month window | 2015–2018 vs 2009–2014 | 20,669 | 677 | 1.000 (0.860–1.164) | 0.996 |
| Surgery | Full follow-up | 2015–2021 vs 2009–2014 | 27,995 | 925 | 0.678 (0.586–0.784) | <0.001 |
| Surgery | Full follow-up, diagnoses through 2018 | 2015–2018 vs 2009–2014 | 21,414 | 874 | 0.698 (0.597–0.815) | <0.001 |
| Surgery | Fixed 36-month window | 2015–2018 vs 2009–2014 | 21,414 | 381 | 0.782 (0.631–0.969) | 0.024 |

*Cardiac death was the event of interest, and all noncardiac deaths were treated as competing events. Models were adjusted for age group, sex, race, summary stage, histological subtype, and primary tumor site. The reference diagnosis period was 2009–2014. sHR, subdistribution hazard ratio; CI, confidence interval.*

## Table S4. Cause-specific Cox sensitivity analyses for cardiac death by cohort and follow-up framework.

| **Cohort** | **Follow-up framework** | **Comparison** | **N** | **Cardiac deaths** | **HR (95% CI)** | **P value** |
| --- | --- | --- | --- | --- | --- | --- |
| Beam radiotherapy | Full follow-up | 2015–2021 vs 2009–2014 | 28,246 | 1,153 | 0.830 (0.734–0.938) | 0.003 |
| Beam radiotherapy | Full follow-up, diagnoses through 2018 | 2015–2018 vs 2009–2014 | 20,669 | 1,022 | 0.860 (0.756–0.979) | 0.023 |
| Beam radiotherapy | Fixed 36-month window | 2015–2018 vs 2009–2014 | 20,669 | 677 | 0.898 (0.770–1.046) | 0.167 |
| Surgery | Full follow-up | 2015–2021 vs 2009–2014 | 27,908 | 925 | 0.818 (0.699–0.956) | 0.012 |
| Surgery | Full follow-up, diagnoses through 2018 | 2015–2018 vs 2009–2014 | 21,369 | 874 | 0.818 (0.693–0.966) | 0.018 |
| Surgery | Fixed 36-month window | 2015–2018 vs 2009–2014 | 21,369 | 381 | 0.753 (0.608–0.933) | 0.010 |

*Cardiac death was the event of interest, and all other deaths were censored. Models were adjusted for age group, sex, race, summary stage, histological subtype, and primary tumor site. Records with unknown race were excluded from the surgery models because that category contained no cardiac deaths and resulted in complete separation; no records were excluded from the radiotherapy models. HR, hazard ratio; CI, confidence interval.*

## Table S5. Diagnosis-period associations in the localized-stage radiotherapy subgroup.

| **Follow-up framework** | **Comparison** | **sHR (95% CI)** | **P value** | **N** | **Cardiac deaths** |
| --- | --- | --- | --- | --- | --- |
| Full follow-up | 2015–2021 vs 2009–2014 | 0.848 (0.735–0.977) | 0.023 | 12,716 | 762 |
| Fixed 36-month window | 2015–2018 vs 2009–2014 | 1.125 (0.920–1.376) | 0.250 | 8,272 | 400 |

*The subgroup was defined by localized summary stage; SEER does not directly record treatment intent. Models were adjusted for age group, sex, race, histological subtype, and primary tumor site, with 2009–2014 as the reference diagnosis period. Cardiac death was the event of interest and death from lung and bronchus cancer the prespecified competing event. sHR, subdistribution hazard ratio; CI, confidence interval.*

## Table S6. Horizon-anchored 36-month cumulative incidence of cardiac death in the radiotherapy cohort.

| **Diagnosis period** | **36-month cardiac CIF** | **Difference (95% CI)** |
| --- | --- | --- |
| 2009–2014 | 3.59% | Reference |
| 2015–2018 | 3.97% | +0.38 percentage points (−0.18 to +0.94) |

*Cumulative incidence was estimated at the fixed 36-month horizon with death from lung and bronchus cancer treated as the prespecified competing event. CIF, cumulative incidence function; CI, confidence interval.*

Figure S1. Covariate balance before and after propensity score matching. Absolute standardized mean differences are shown for covariates included in the propensity score model. Open circles indicate the original unmatched cohort, filled squares indicate the propensity score-matched cohort, and line segments connect the before and after matching values for each covariate. The dashed vertical line denotes the prespecified balance target of an absolute standardized mean difference of 0.10. Diagnosis period was not included in the propensity score model and is therefore not displayed.
